# Resection of a schwannoma originating from vagus nerve in the right superior mediastinum

**DOI:** 10.1186/s13019-023-02177-6

**Published:** 2023-02-10

**Authors:** Ming Zhang, Hao Shi, Xiabin Tu, Weidong Li, Zhengliang Wei

**Affiliations:** 1 Department of Cardiothoracic Surgery, Shengzhou People’s Hospital (The First Affiliated Hospital of Zhejiang University Shengzhou Branch), Shaoxing, 312400 China; 2 Department of Cardiovascular Surgery, The First Affiliated Hospital, School of Medicine, Zhejiang University, Hangzhou, 310003 China

**Keywords:** Schwannoma, Mediastinal neurogenic tumor, Vagus nerve

## Abstract

**Background:**

Schwannoma is the most common mediastinal neurogenic tumor, while schwannoma originating from mediastinal vagus nerve is rare.

**Case presentation:**

This article reported one case of schwannoma originating from vagus nerve in the right superior mediastinum. The mediastinal schwannoma was completely resected through a right-sided video-assisted thoracoscopic thoracotomy. Histologic examination clarified the diagnosis as schwannoma.

**Conclusion:**

Chest CT scan and MRI can be used to determine the location of mediastinal schwannoma and its relationship with adjacent tissue. Histologic examination showing distinctive feature of Antoni A areas and Antoni B areas can help clarify the diagnosis. Complete surgical resection is the first-line treatment option for mediastinal schwannomas.

## Background

Schwannoma is a benign tumor originating from the neural sheath Schwann cells, with approximately 9% of which occurring in the mediastinum [1]. Schwannoma is the most common mediastinal neurogenic tumor, which accounts for 25.3% of intrathoracic neurogenic tumors [2]. Nearly 10% of schwannomas originate from vagus nerve [3], while schwannoma originating from mediastinal vagus nerve is rare. In this article, we reported one case of schwannoma originating from vagus nerve in the right superior mediastinum.

## Case presentation

**Fig. 1:**
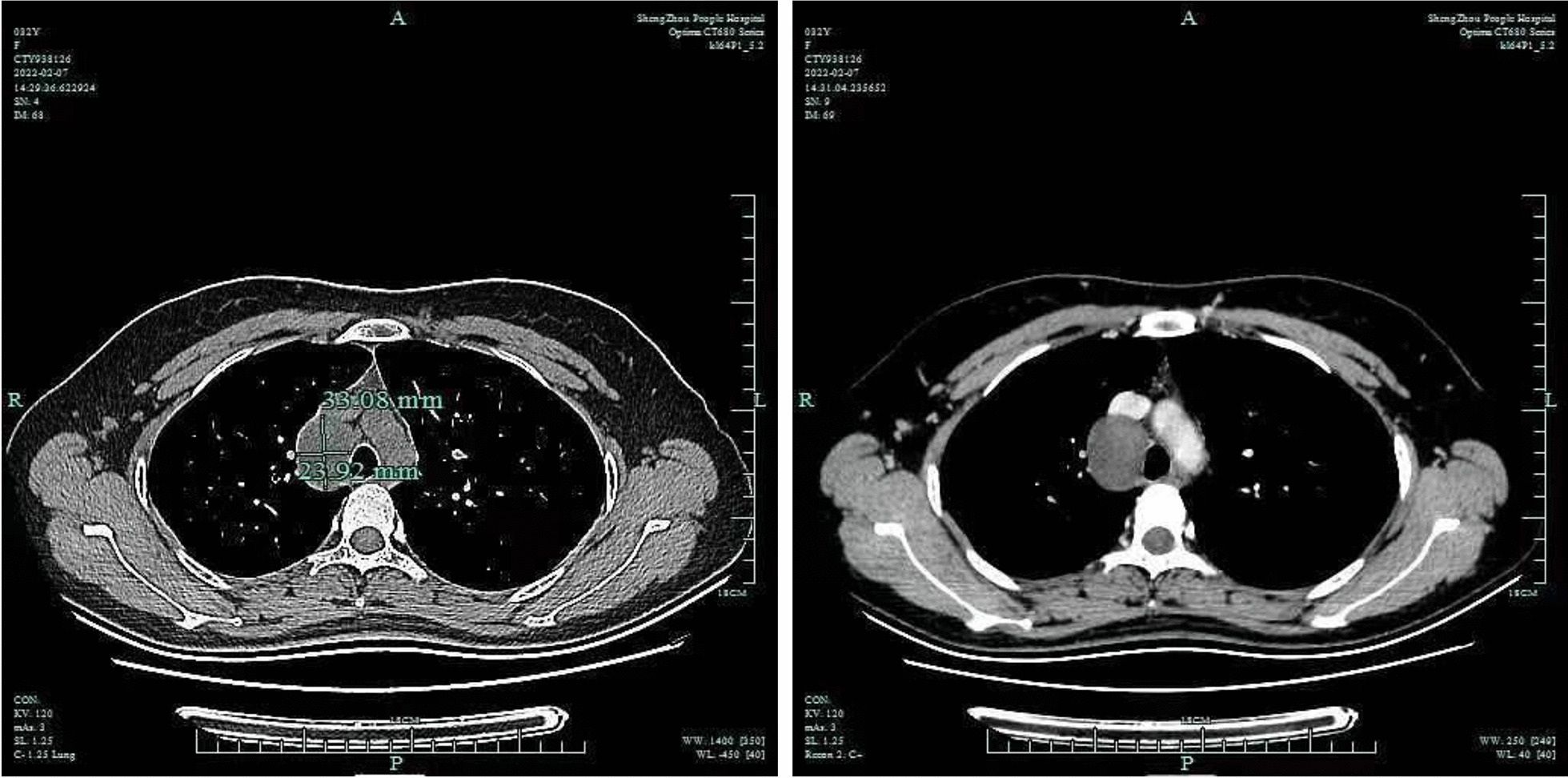
Contrast-enhanced chest CT scan revealed a sharply marginated mass, 24 × 33 mm in size, in the right superior mediastinum

**Fig. 2:**
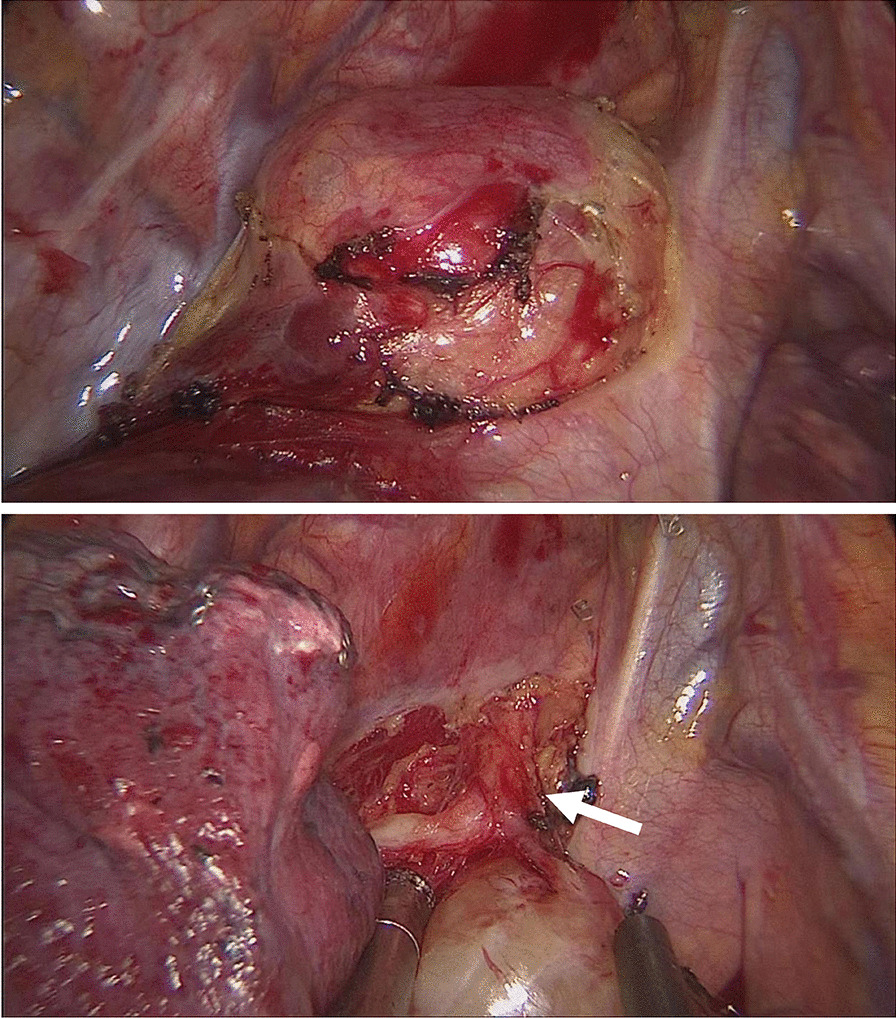
Intraoperative view of the mediastinal schwannoma originating from vagus nerve (marked by white arrow)

**Fig. 3:**
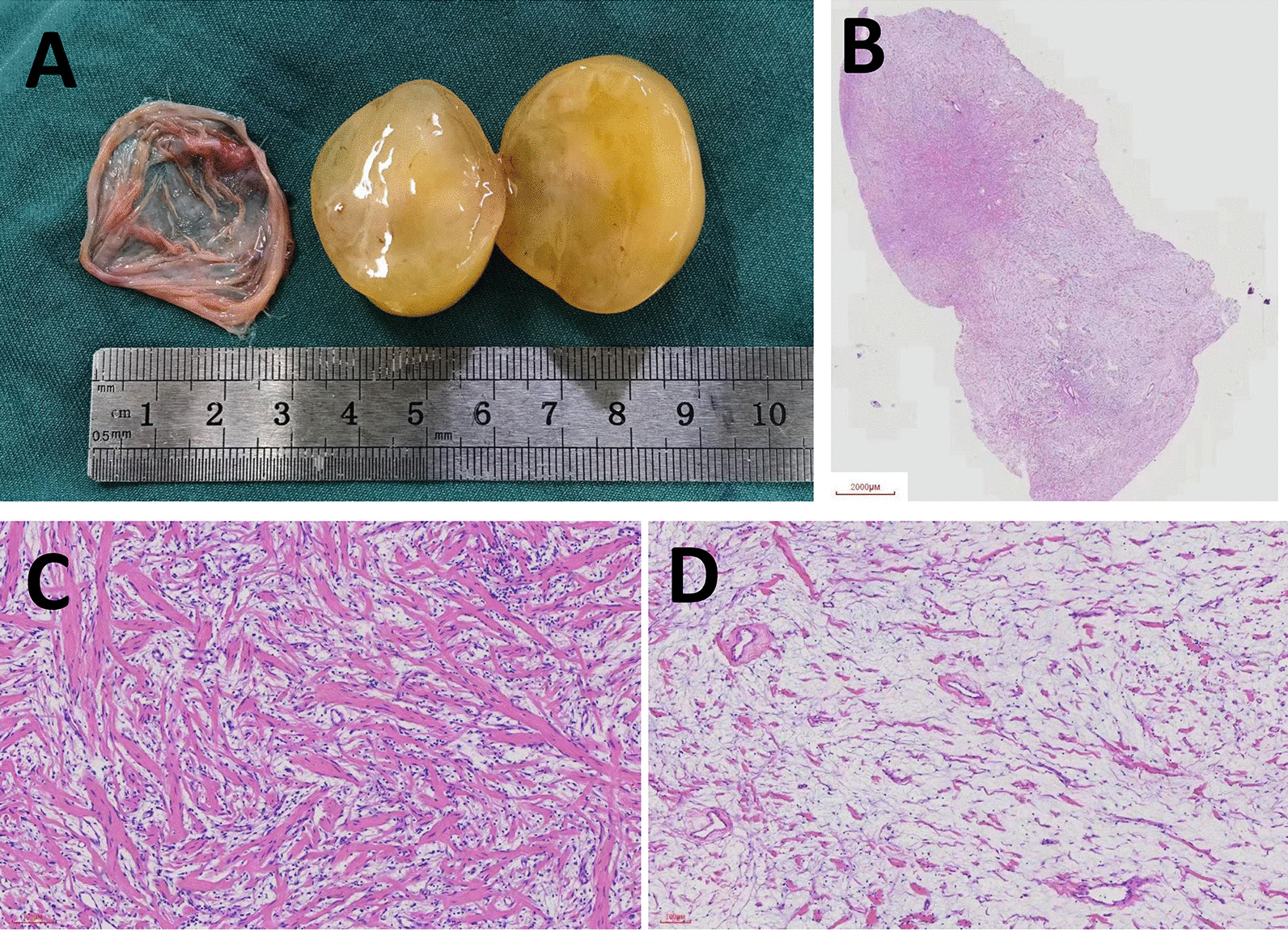
Gross morphology of the mediastinal schwannoma (**A**). Histologic examination of the mediastinal schwannoma (**B**–**D**). The tumor was formed by spindle cells (**C**) and loose myxoid tissue (**D**), which clarified the diagnosis as schwannoma

A 32-year-old asymptomatic woman with a mediastinal mass found by CT scan 2 years ago was admitted to our hospital. Physical examination and laboratory tests showed no significant abnormalities. Contrast-enhanced chest CT scan revealed a sharply marginated mass, 24 × 33 mm in size, in the right superior mediastinum (Fig. 1). A right-sided video-assisted thoracoscopic thoracotomy was performed, a 3 cm uniport through the 4th intercostal at midaxillary line was made to perform the procedure. Intraoperatively, a 4 × 3 cm mass was found in the right superior mediastinum, which was originated from the right vagus nerve. The boundary of the tumor was clear, and the tumor was excised completely (Fig. 2). Grossly, the tumor was encapsulated by a complete envelope, with yellow-colored solid component inside (Fig. 3A). Histologic examination revealed that the tumor was formed by spindle cells and loose myxoid tissue, which clarified the diagnosis as schwannoma (Fig. 3B–D). The patient recovered uneventfully after the surgery, and was discharged on the 3rd postoperative day.

## Discussion

Mediastinal schwannomas usually occur in the posterior mediastinum arising from intercostal nerve, sympathetic chain or posterior root of spinal nerve, while those originating from intrathoracic vagus nerve are rare [4]. When these tumors compress the adjacent structures, they will cause symptoms such as chest pain, hoarseness, dyspnea, or dysphagia [5, 6]. Other rare symptoms caused by mediastinal schwannomas include inappropriate secretion of antidiuretic hormone syndrome (IADH syndrome) [7], Horner syndrome [8], intrathoracic bleeding [9], and pleural effusion [10].

Mediastinal schwannomas present grossly as solitary and well-encapsulated tumors, which have a sharp border with the adjacent soft tissue. Nerve of origin usually present at the periphery of the tumor, instead of penetrating the tumor. CT scan can be used to determine the location of mediastinal schwannoma and its relationship with adjacent tissue. MRI can help better determine the relationship of a mediastinal schwannoma with adjacent vascular structures [11]. Under microscopic examination, mediastinal schwannomas are composed of two histological components: Antoni A areas and Antoni B areas. The Antoni A area is formed by densely arranged spindle cells, and the Antoni B area is formed by loose myxoid tissue, which often develops mucoid degeneration, cystic change or hemorrhage. Immunohistochemical staining of the spindle cells in schwannomas is positive for S-100 protein in cytoplasm, and positive for SOX-10 in nuclear [6].

There are several special variants of schwannoma that have been described in the mediastinum. Ancient schwannomas have a high frequency of regressive changes, such as fatty degeneration, cystic formation, hemorrhage, and calcification, which may grow to unusually large size. This variant of schwannoma still maintains its strong diffuse S-100 positivity by Immunohistochemistry, which helps distinguish it from malignant schwannomas [9]. Melanotic schwannomas are made up of Schwann cells, with capacity for melanogenesis. This variant of schwannoma contains melanosomes in different stage of maturation [12]. Malignant transformation of schwannomas usually occurs to malignant peripheral nerve sheath tumor (MPNST), angiosarcoma or epithelioid malignant change. Malignant schwannoma can exhibit other cellular components such as epithelial cells or mesenchymal features [11].

Complete surgical resection is the first-line treatment option for mediastinal schwannomas. Video-assisted thoracoscopy (VATS) technique has been gradually accepted as a safe and reliable access for resection of mediastinal schwannomas, which can reduce surgical trauma, accelerate postoperative recovery, while permit good exposure of the mediastinum. However, for tumors with more than 6 cm in size, or located at the costophrenic angle or thoracic apex, open approaches such as thoracotomy, sternotomy, supraclavicular excision or posterolateral thoracotomy are more appropriate [13]. It has been reported that preoperative angiography embolization could reduce tumor vascularity and operative blood loss, which facilitated the resection of large mediastinal schwannomas [14]. Intraoperative neurostimulation and neuromonitoring approach were recommended to reduce the risk of nerve injury [15].

In this case, the essential of the operation was to expose the tumor clearly and to resect it completely, without injuring the vagus nerve. In order to achieve those purposes, we chose a 3 cm uniport through the 4th intercostal at midaxillary line to perform the video-assisted thoracoscopic thoracotomy. Electrocantery and aspirator were used to separate the capsule of the tumor and expose the vagus nerve clearly. After the pedicle of the tumor was adequately exposed, ultrasonic scalpel was used to divide the pedicle and complete the resection.

## Conclusions

Schwannoma is a benign tumor originating from the neural sheath Schwann cells, which is the most common mediastinal neurogenic tumor, while schwannoma originating from mediastinal vagus nerve is rare. Chest CT scan and MRI can be used to determine the location of mediastinal schwannoma and its relationship with adjacent tissue. Histologic examination showing distinctive feature of Antoni A areas and Antoni B areas can help clarify the diagnosis. Complete surgical resection is the first-line treatment option for mediastinal schwannomas.

## Data Availability

Please contact author for data requests.

## Abbreviations

IADH syndrome: Inappropriate secretion of antidiuretic hormone syndrome
MPNST: Malignant peripheral nerve sheath tumor
VATS: Video-assisted thoracoscopy
